# Concentric hip work is the most robust lower-limb kinetic correlate of depth-jump height in male collegiate volleyball players

**DOI:** 10.1186/s13102-026-01968-3

**Published:** 2026-08-11

**Authors:** Di Lu, Cheng Chen, Yueming Li, Wenfeng Zhang, Jian Sun, Duanying Li

**Affiliations:** 1 School of Physical Education, Guangzhou Sport University, Guangzhou, China; 2 Sports Training Institute, Guangzhou Sport University, Guangzhou, Guangdong China; 3 Key Laboratory of Human-Computer Intelligent Interaction for Athletic Performance and Health Promotion, Guangzhou Sport University, Guangzhou, Guangdong China; 4 Guangdong Provincial Key Laboratory of Human Sports Performance Science, Guangzhou Sport University, Guangzhou, Guangdong China

**Keywords:** Volleyball, Depth jump, Movement analysis, Sports performance, Joint kinetics, Hip work

## Abstract

**Background:**

Depth-jump height is commonly used to assess lower-limb explosive performance, but it remains unclear which phase-specific hip, knee, and ankle joint kinetic variables are most consistently associated with performance in volleyball players.

**Methods:**

In this cross-sectional movement-analysis study, thirty male collegiate volleyball players completed height-oriented depth-jump tests from a standardized 30-cm box. Three-dimensional motion capture and two force platforms were used to quantify jump height, hip, knee, and ankle joint stiffness, and peak joint moment, peak joint power, and joint work during the ground-contact braking/eccentric and propulsive/concentric phases. Participants were ranked by jump height and divided into a high-jump group (HJG) and a low-jump group (LJG) using a median split for descriptive comparison, whereas Pearson correlation analysis across the full sample was used as the primary continuous analysis. Benjamini-Hochberg false discovery rate (FDR) correction was applied to account for multiple comparisons.

**Results:**

HJG achieved greater jump height than LJG. In uncorrected analyses, HJG showed higher concentric-phase peak hip moment, peak hip power, hip joint work, and ankle joint work. After FDR correction, only concentric-phase hip joint work remained significantly higher in HJG than in LJG (*p* < 0.001, q = 0.012). Correlation analysis showed that concentric-phase hip joint work had the strongest association with jump height (*r* = 0.610, 95% CI: 0.320 to 0.795, *p* < 0.001, q = 0.007). Concentric-phase peak hip moment, peak hip power, and ankle joint work showed uncorrected positive associations, but these findings did not survive FDR correction.

**Conclusions:**

Among the variables examined, greater depth-jump height in male collegiate volleyball players was most robustly associated with cumulative positive hip work during the concentric propulsive phase. Supportive uncorrected findings for peak hip moment, peak hip power, and ankle joint work should be interpreted as exploratory. Concentric hip work may be useful as an associative monitoring variable and a candidate for longitudinal mechanistic testing, but the present cross-sectional findings do not establish that increasing hip work will cause improvements in depth-jump height.

**Supplementary Information:**

The online version contains supplementary material available at https://doi.org/10.1186/s13102-026-01968-3.

## Background

Vertical jumping ability is a fundamental physical quality for volleyball players because spiking, blocking, and many setting actions require repeated jumps under time pressure. During international volleyball rallies, players are required to perform rapid jumping tasks in short sequences, and front-row players may perform several block and spike jumps within a single rally [1]. Volleyball athletes also need to reach maximal or near-maximal jump heights during decisive offensive and defensive actions [2]. Martinez [3] further emphasized that vertical jump capacity is directly relevant to power production and technical effectiveness in volleyball.

Previous research has shown that jump performance varies with competitive level in volleyball. Reviews and cross-sectional comparisons of professional divisions and junior national, state, and novice cohorts have reported differences in vertical-jump performance and lower-body muscular power across levels of play [2, 4–6]. Tournament-level evidence also indicates that player characteristics, including jumping ability, may be related to team success in national competition [7]. Explosive-strength development is therefore a central component of volleyball training programs designed to improve court performance [8, 9].

The depth jump is widely used in both plyometric training and lower-limb performance testing. Unlike a countermovement jump performed from a standing start, the depth jump begins with a drop from a box and is followed by landing, braking, and a subsequent vertical take-off. Bobbert et al. [10] described the depth jump as a task that differs mechanically from the countermovement jump because the athlete must manage landing impact before producing upward propulsion. Larger stretch amplitudes during drop-jump tasks can alter neuromuscular activity and change the mechanical demands imposed on the lower limbs [11]. At the same time, the depth jump can enhance vertical jump potentiation by combining landing-induced loading with powerful concentric propulsion [12].

From a biomechanical perspective, the complete depth-jump task comprises the step-off and downward-flight phase, initial ground contact, a braking phase in which the body center of mass continues to move downward, the transition at minimum center-of-mass height, a propulsive phase in which the center of mass moves upward, take-off, and the subsequent flight phase [10, 13]. During braking, the lower-limb joints absorb mechanical energy, whereas during propulsion they generate net positive joint work to accelerate the body center of mass upward. Muscle-tendon behavior during jumping is closely related to energy storage and release [14], and the kinetic behavior of the hip, knee, and ankle contributes to rebound- and vertical-jump performance [15, 16]. Because the present study quantified joint kinetics during ground contact, the inferential analyses focused on the braking/eccentric and propulsive/concentric phases.

Previous studies have related rebound- and vertical-jump performance to lower-limb joint moments, power, work, and inter-joint coordination [15–19]. Other investigations have shown that verbal instructions, countermovement range of motion, and the selected jumping strategy can materially alter lower-limb kinematic and kinetic profiles [20–22]. However, the phase-specific relationships between depth-jump height and hip, knee, and ankle kinetic variables remain insufficiently characterized in volleyball players. This distinction is important because the braking phase reflects landing control, energy absorption, and preparation for propulsion, whereas the propulsive phase more directly reflects the net mechanical work used to generate upward center-of-mass velocity. Clarifying whether depth-jump height is more strongly associated with braking- or propulsive-phase kinetic variables may improve the interpretation of biomechanical assessments and help identify variables for subsequent longitudinal testing.

Therefore, this study aimed to examine phase-specific lower-limb kinetic variables associated with depth-jump height in male collegiate volleyball players. By combining a descriptive comparison between higher and lower jump-height performers with continuous correlation analysis across the full sample, this study sought to determine whether greater depth-jump height was associated with a specific phase- and joint-dependent kinetic pattern rather than a uniform increase in all lower-limb kinetic variables. We hypothesized that athletes with greater depth-jump height would show higher concentric-phase lower-limb kinetic output, particularly at the hip and knee, and that concentric-phase kinetic variables would be positively associated with jump height.

## Methods

### Experimental approach to the problem

This cross-sectional biomechanical study investigated whether depth-jump height was associated with phase-specific hip, knee, and ankle kinetic variables in male collegiate volleyball players. In the present study, the term depth jump refers to a drop from a fixed 30-cm box followed immediately by a maximal vertical jump. Thirty athletes completed testing during a 3-day data-collection period. Kinematic and kinetic data were collected during the ground-contact braking/eccentric and propulsive/concentric phases, and jump height was calculated for each participant.

**Fig. 1:**
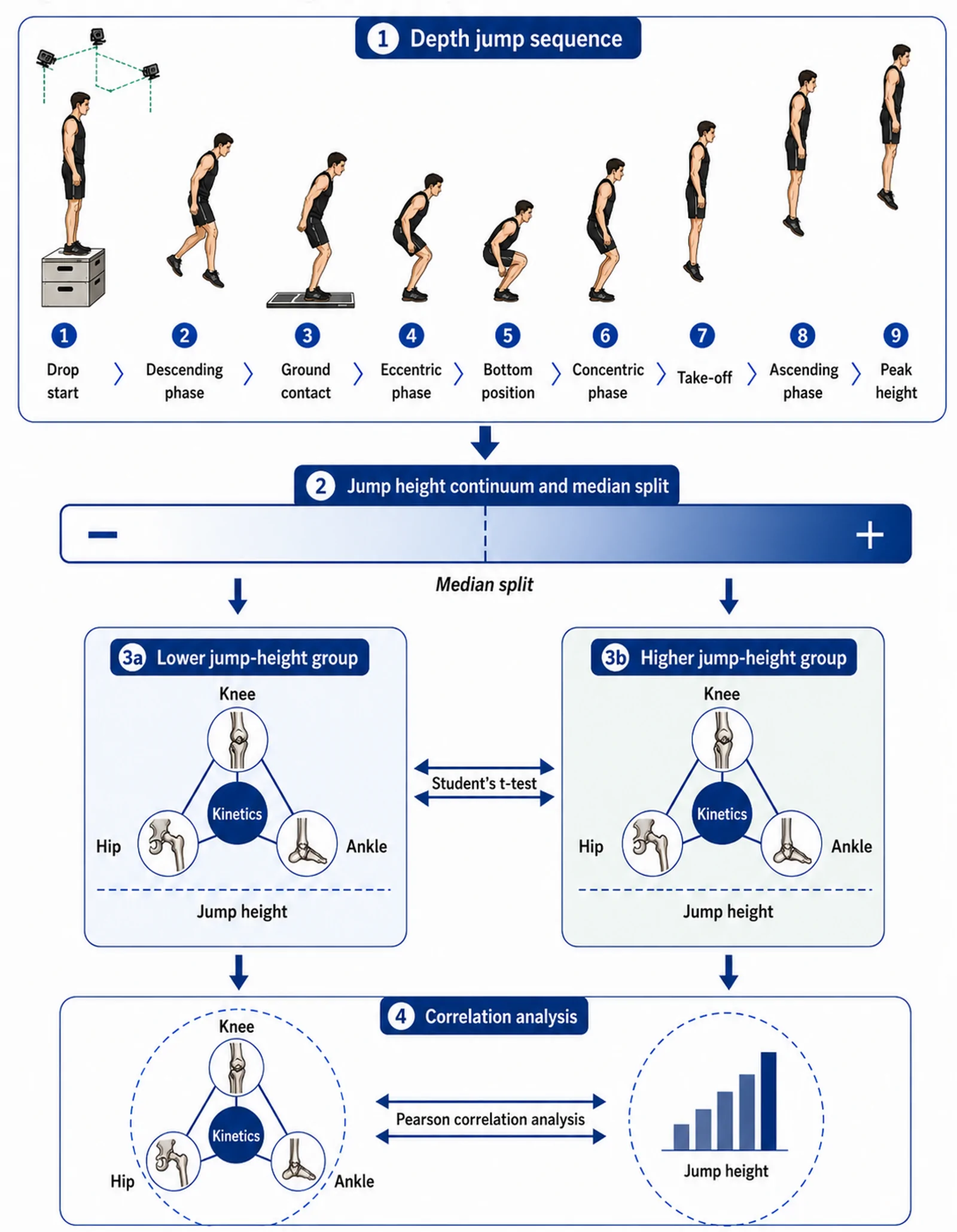
Research design and analytical workflow

Participants were ranked from highest to lowest according to depth-jump height. A median split was used to classify the 15 athletes with higher jump height as HJG and the remaining 15 athletes as LJG. This grouping was used only to provide an interpretable descriptive comparison between relatively higher and lower performers. The primary interpretation of the relationship between kinetic variables and jump height was based on Pearson correlation analysis across the full sample because jump height is a continuous performance outcome and dichotomization reduces information from the original variable (Fig. 1).

### Participants

Thirty male collegiate volleyball players volunteered to participate in the study. Eligibility required at least 4 years of systematic volleyball-specific training, current participation in three structured technical and tactical training sessions per week of approximately 2 h each (approximately 6 h per week), and at least 4 years of structured resistance training that included plyometric, strength, and other physical-conditioning exercises. All participants held at least National Second-Class Athlete certification in China and had competed in at least one Chinese provincial-level event, including the Provincial Games, Provincial Volleyball Championships, or Provincial Collegiate Volleyball League. Participants were not included if they reported current lower-limb injury or pain that limited maximal jumping, were unable to complete the testing protocol safely, or produced signals that could not be processed reliably. There were no significant differences between HJG and LJG in age, height, body mass, or training experience (Table 1). Before testing, all participants were informed of the potential risks of the experiment and signed written informed consent. The study was conducted in accordance with the Declaration of Helsinki and was approved by the Ethics Committee of Guangzhou Sport University (approval number: 2025LCLL-001).

**Table 1:** Participant characteristics

Variable	HJG (*n* = 15)	LJG (*n* = 15)	Total (*n* = 30)
Age (years)	19.7 ± 0.9	19.6 ± 1.0	19.6 ± 0.9
Height (cm)	185.1 ± 4.7	187.1 ± 5.8	186.1 ± 5.2
Body mass (kg)	75.5 ± 6.7	80.2 ± 11.8	77.9 ± 9.7
Training experience (years)	5.6 ± 1.9	6.9 ± 2.6	6.3 ± 2.4

*HJG *high-jump group, *LJG *low-jump group

Values are mean ± SD

### Procedures

Data collection for the full sample was completed over 3 days, and participants avoided high-intensity physical training for 48 h before testing. Three-dimensional kinematic data were collected at 200 Hz using a 10-camera Qualisys optical motion-capture system operated with Qualisys Track Manager 2023.3 (build 12256; Qualisys AB, Gothenburg, Sweden). Ground-reaction-force data were collected synchronously at 1000 Hz using two Bertec FP4060-08-TM-2000 force platforms (Bertec Corporation, Columbus, OH, USA). Kinematic and kinetic data were subsequently processed using Visual3D (C-Motion Inc., Germantown, MD, USA). The motion-capture volume and force platforms were calibrated according to the manufacturers’ procedures before data collection. After arriving at the laboratory and changing clothes, participants completed a standardized warm-up led by the same investigator. The warm-up included soft-tissue preparation of major muscle groups, dynamic stretching such as walking quadriceps stretches, neural activation drills such as fast high-knee running, and movement-integration exercises such as squats. The same warm-up sequence and testing environment were used for all participants.

After the warm-up, a 59-marker whole-body CAST configuration was attached for the static calibration trial. The configuration included markers on the head, trunk, bilateral upper limbs, pelvis, thighs, shanks, and feet, together with four auxiliary markers used in the laboratory model. Four-marker tracking clusters were positioned on each thigh and shank. The anatomical coordinate-system definitions followed the recommendations of Cappozzo et al. [23], and anatomical landmarks were identified according to Van Sint Jan [24]. A standardized standing trial was recorded to construct the participant-specific model. After static calibration, eight anatomical calibration markers located around the bilateral knees and ankles were removed, leaving 51 tracking markers for the dynamic depth-jump trials. Marker placement was performed by the same investigator throughout testing.

**Fig. 2:**
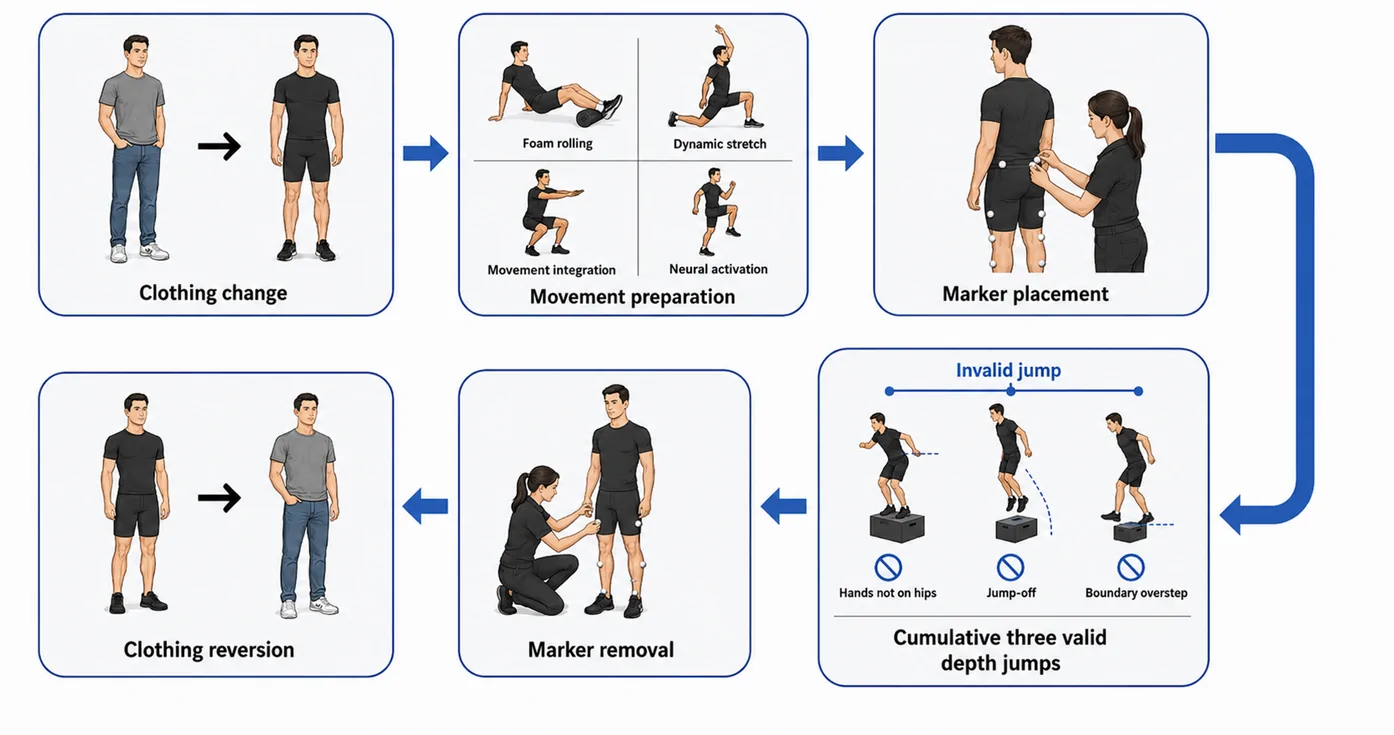
Experimental testing procedure

Before formal data collection, participants completed a supervised familiarization procedure comprising light whole-body activity, dynamic lower-limb mobility exercises, submaximal vertical jumps, and practice depth jumps from the 30-cm box. Familiarization was criterion-based rather than prescribed as a fixed number of attempts or sets. Practice was performed at light-to-moderate intensity and continued until the testing team judged that the participant could safely and consistently step from the box, land bilaterally with one foot on each force platform, maintain the hands-on-hips position, and complete the subsequent vertical jump without loss of balance. No separate multi-day familiarization program was used. A 30-cm drop height was selected as a standardized height commonly used in trained athletes and skilled volleyball players and to avoid the greater impact loading and altered movement strategies that can occur at higher drop heights [20, 25, 26]. Participants were allowed to step off the box with either foot and fall freely, but they were not allowed to jump upward from the box. During the entire depth jump, participants kept their hands on their hips to limit arm-swing contribution and were required to land bilaterally with one foot fully on each force platform. They were repeatedly instructed to jump as high as possible. The protocol therefore represented a height-oriented depth jump with a self-selected countermovement strategy rather than a minimum-contact-time or fast stretch-shortening-cycle test. Ground contact time and countermovement amplitude were not restricted, and trials were not excluded or analyzed separately solely because of a longer ground contact time or deeper countermovement. A trial was repeated if the participant used an arm swing, jumped upward from the box, failed to land cleanly on the force platforms, lost balance, or produced signals that prevented reliable event detection. Each participant completed three valid depth jumps, with 90 s of rest between trials. The mean value of the three valid trials was used for participant-level analysis (Fig. 2).

### Data processing

Depth-jump kinematic and kinetic data were smoothed using a fourth-order recursive Butterworth low-pass filter with a 15-Hz cut-off frequency. The same cut-off frequency was applied to marker and force-platform data to retain the primary movement signal during the short ground-contact phase while attenuating high-frequency noise. This filtering approach is consistent with previous drop vertical-jump biomechanical assessments [20]. The ground-contact period was divided according to the vertical velocity of the body center of mass. The braking/eccentric phase extended from initial contact until vertical center-of-mass velocity reached zero at minimum center-of-mass height, and the propulsive/concentric phase extended from this transition until take-off [13]. These phase labels describe the direction of whole-body center-of-mass motion and the predominant net mechanical function of the lower limbs; they do not imply uniform muscle action at every joint throughout each phase.

Vertical ground-reaction forces from the two force platforms were summed for whole-body event detection. Ground contact began when the summed vertical ground reaction force first reached at least 10 N. Take-off was identified when summed vertical ground reaction force decreased to 10 N or less, following the threshold approach used by Zhang et al. [27]. Peak joint moment, peak joint power, and joint work of the hip, knee, and ankle during the eccentric and concentric phases were calculated using inverse dynamics. For each valid trial, left- and right-limb joint-level variables were averaged to obtain bilateral hip, knee, and ankle values. Joint power was calculated from joint moment and joint angular velocity, and joint work was calculated as the time integral of joint power within each phase. For ease of interpretation, eccentric-phase work was expressed as the absolute magnitude of negative work, whereas concentric-phase work represented positive work output. Joint moment, power, and work variables were normalized to body mass.

Joint stiffness was calculated as the peak joint moment during ground contact divided by the maximum change in joint angle during ground contact. This definition follows the lower-limb stiffness framework summarized by Maloney and Fletcher [28]. Peak angular velocity and peak flexion angle at the hip, knee, and ankle were also extracted to provide kinematic context for the between-group comparison figure. Jump height was calculated as the difference between the maximum center-of-mass height during the take-off phase and the center-of-mass height in the standardized standing position. This center-of-mass displacement approach is consistent with the jump-height calculation methods reviewed by Xu [29].

### Statistical analysis

Statistical analyses were conducted using R and SPSS 27.0. The Shapiro-Wilk test was used to assess the normality of each variable. Variables violating the normality assumption were transformed using the Box-Cox procedure before inferential testing [30]. In the final analysis, Box-Cox transformation was applied to eccentric-phase ankle joint work and hip, knee, and ankle stiffness; descriptive statistics are presented as raw mean ± SD values, whereas inferential tests were conducted using transformed values when appropriate. Detailed normality and transformation results are provided in Additional file 1. Median-split comparisons between HJG and LJG were treated as descriptive contrasts only because the groups were created from the jump-height outcome and dichotomization reduces continuous information. Continuous correlation analysis across all participants was therefore treated as the primary analysis of the relationship between kinetic variables and depth-jump height.

Welch’s independent-samples t-tests were used for median-split comparisons between HJG and LJG to reduce sensitivity to unequal variances. Hedges’ g was calculated from raw participant-level values to quantify the magnitude of between-group differences while preserving interpretability. Effect sizes were interpreted using the thresholds reported by McGrath and Meyer [31]: very small (< 0.2), small (0.2 to < 0.5), moderate (0.5 to < 0.8), and large ( > = 0.8). Pearson correlation coefficients were used to examine associations between each kinetic variable and jump height across all participants, and 95% confidence intervals for r were calculated using Fisher’s z transformation. Correlation magnitudes were interpreted according to Schober et al. [32]. To account for multiple comparisons, Benjamini-Hochberg FDR correction was applied separately to the 21 between-group comparisons and the 21 correlation analyses. Both uncorrected p values and FDR-adjusted q values are reported. Corrected statistical significance was set at q < 0.05, whereas findings with *p* < 0.05 but q > = 0.05 were interpreted as exploratory. A sensitivity power analysis indicated that the available sample could detect a large between-group effect of approximately Cohen’s d = 1.06 and a Pearson correlation of approximately |r| = 0.492 with 80% power at alpha = 0.05; the full statistical output is provided in Additional file 1.

## Results

**Fig. 3:**
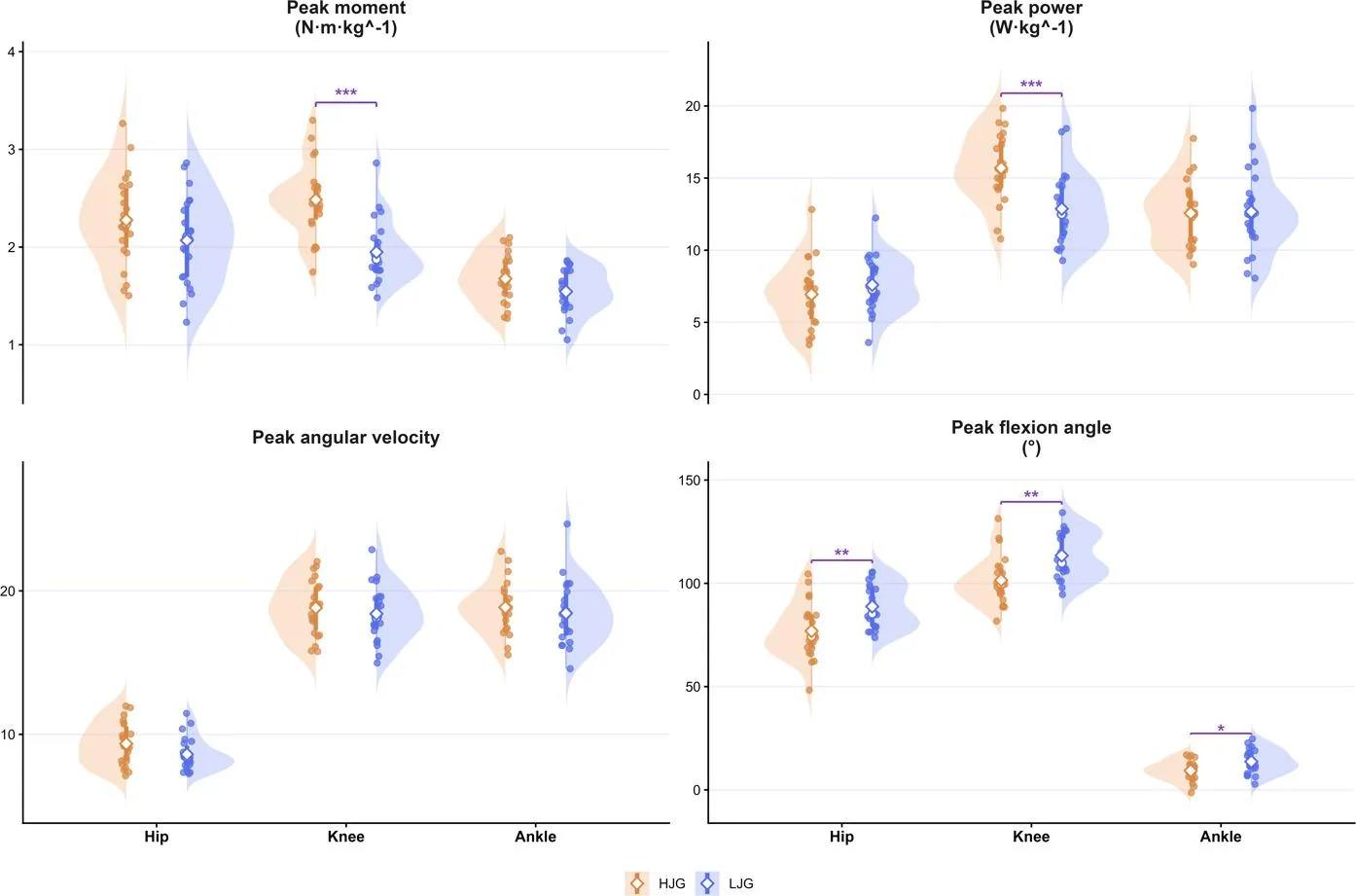
Between-group differences in hip, knee, and ankle peak moment, peak power, peak angular velocity, and peak flexion angle between HJG and LJG. Data are shown as individual values and distribution plots. Asterisks indicate uncorrected p values shown for visual reference only; FDR-adjusted q values for primary kinetic and stiffness variables are reported in Table 2. The kinematic variables are presented as descriptive movement context and were not included in the primary inferential family. *p < 0.05, **p < 0.01, ***p < 0.001

**Fig. 4:**
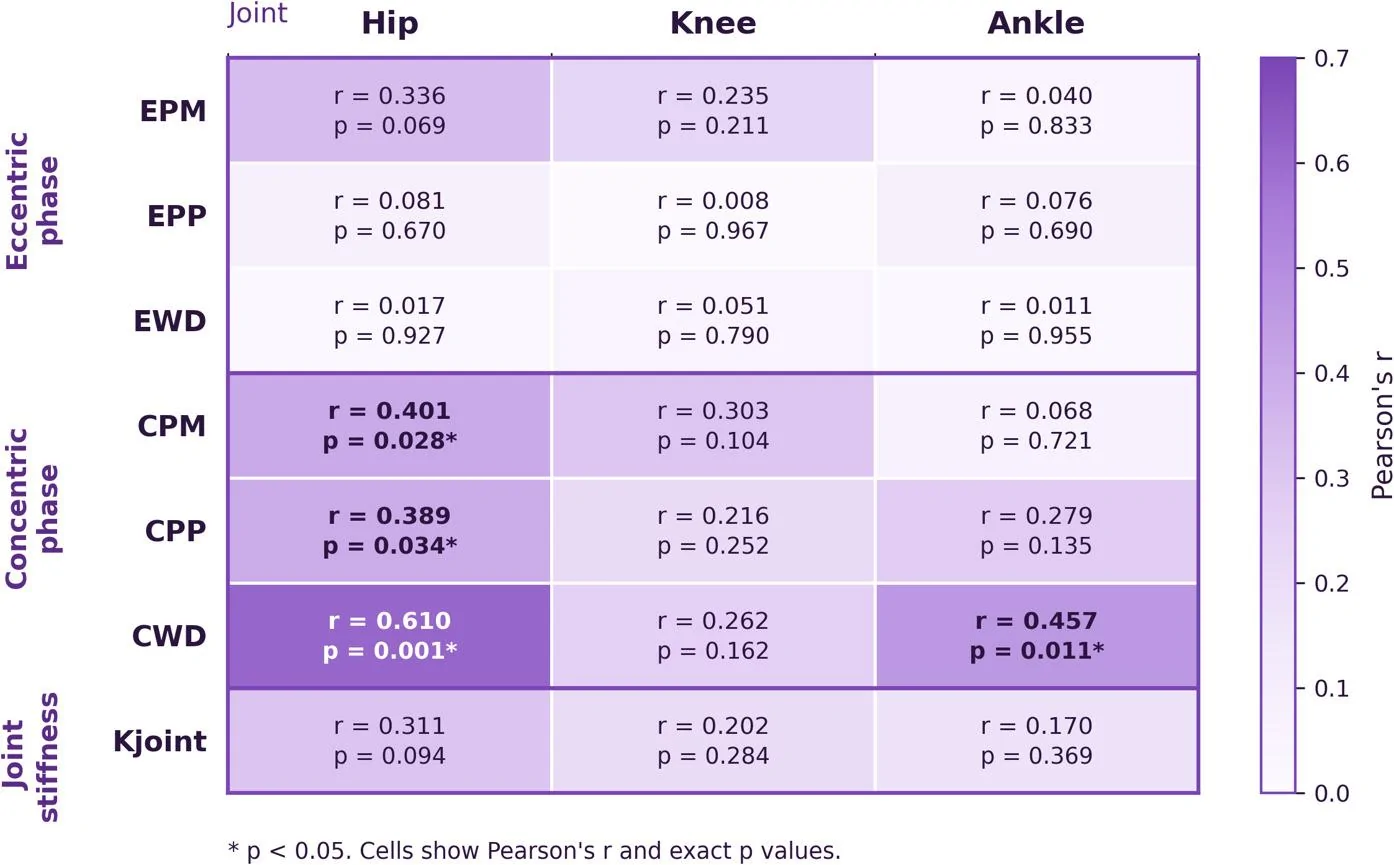
Correlations between hip, knee, and ankle joint kinetics and jump height. Cells show Pearson’s r and uncorrected p values; FDR-adjusted q values are reported in Table 3. EPM = eccentric peak moment; EPP = eccentric peak power; EWD = eccentric work done; CPM = concentric peak moment; CPP = concentric peak power; CWD = concentric work done; Kjoint = joint stiffness. *p < 0.05

As expected from the grouping procedure, HJG achieved greater depth-jump height than LJG; this contrast was descriptive and was not interpreted as independent confirmatory evidence. Group comparisons indicated that the clearest kinetic differences were concentrated in the concentric phase. In uncorrected analyses, HJG showed higher concentric-phase peak hip moment (*p* = 0.025, Hedges’ g = 0.843), peak hip power (*p* = 0.022, Hedges’ g = 0.864), hip joint work (*p* < 0.001, Hedges’ g = 1.381), and ankle joint work (*p* = 0.037, Hedges’ g = 0.777). After FDR correction, only concentric-phase hip joint work remained statistically significant (q = 0.012). The uncorrected differences in peak hip moment, peak hip power, and ankle joint work showed moderate-to-large effects but did not survive FDR correction and should therefore be interpreted as exploratory. Figure 3 provides a descriptive visualization of selected joint-level variables. The kinematic variables shown in Fig. 3 were included only to contextualize the movement pattern and were not part of the primary inferential family; the inferential interpretation of group differences should be based on Table 2 (Fig. 4).

**Table 2:** Hip, knee, and ankle joint kinetics in HJG and LJG

Variable	HJG	LJG	*p*	q	Hedges’ g (95% CI)
Eccentric phase					
Peak hip moment	2.521 ± 0.591	2.136 ± 0.561	0.077	0.315	0.652 (-0.084, 1.387)
Peak hip power	14.925 ± 6.327	13.437 ± 7.778	0.570	0.665	0.204 (-0.513, 0.922)
Hip joint work	0.704 ± 0.290	0.566 ± 0.331	0.234	0.452	0.432 (-0.293, 1.157)
Peak knee moment	2.669 ± 0.468	2.374 ± 0.460	0.092	0.315	0.619 (-0.115, 1.353)
Peak knee power	16.120 ± 3.940	14.197 ± 6.464	0.333	0.479	0.350 (-0.372, 1.071)
Knee joint work	1.351 ± 0.289	1.187 ± 0.492	0.278	0.479	0.393 (-0.330, 1.116)
Peak ankle moment	1.373 ± 0.278	1.289 ± 0.257	0.399	0.523	0.305 (-0.416, 1.025)
Peak ankle power	5.670 ± 2.950	5.326 ± 2.212	0.720	0.720	0.128 (-0.588, 0.845)
Ankle joint work	0.432 ± 0.203	0.392 ± 0.158	0.626	0.692	0.218 (-0.500, 0.936)
Concentric phase					
Peak hip moment	2.402 ± 0.489	1.998 ± 0.445	0.025	0.174	0.843 (0.094, 1.591)
Peak hip power	9.015 ± 2.509	6.946 ± 2.137	0.022	0.174	0.864 (0.113, 1.614)
Hip joint work	1.004 ± 0.301	0.612 ± 0.250	< 0.001	0.012	1.381 (0.579, 2.183)
Peak knee moment	2.585 ± 0.547	2.294 ± 0.392	0.105	0.315	0.595 (-0.137, 1.328)
Peak knee power	14.421 ± 3.035	13.479 ± 1.879	0.315	0.479	0.363 (-0.359, 1.085)
Knee joint work	1.583 ± 0.321	1.463 ± 0.187	0.222	0.452	0.444 (-0.281, 1.169)
Peak ankle moment	1.710 ± 0.229	1.620 ± 0.276	0.342	0.479	0.343 (-0.378, 1.065)
Peak ankle power	12.925 ± 2.669	11.742 ± 1.943	0.176	0.452	0.493 (-0.234, 1.220)
Ankle joint work	1.090 ± 0.203	0.954 ± 0.127	0.037	0.196	0.777 (0.033, 1.521)
Joint stiffness					
Hip stiffness	0.076 ± 0.035	0.065 ± 0.037	0.237	0.452	0.287 (-0.432, 1.007)
Knee stiffness	0.062 ± 0.044	0.059 ± 0.054	0.703	0.720	0.048 (-0.668, 0.764)
Ankle stiffness	0.038 ± 0.017	0.036 ± 0.020	0.498	0.616	0.113 (-0.603, 0.829)

Moment, power, work, and stiffness are expressed as N*m*kg^-1, W*kg^-1, J*kg^-1, and N*m*rad^-1*kg^-1, respectively

*HJG* high-jump group, *LJG* low-jump group

Values are mean ± SD

p values are uncorrected; q values are Benjamini-Hochberg FDR-adjusted values across the 21 kinetic and stiffness comparisons. Box-Cox transformed variables are reported in Additional file 1

Pearson correlation analysis showed a parallel pattern. Depth-jump height was most strongly associated with concentric-phase hip joint work (*r* = 0.610, 95% CI: 0.320 to 0.795, *p* < 0.001, q = 0.007), and this association remained significant after FDR correction. Additional uncorrected positive associations were observed for concentric-phase peak hip moment (*r* = 0.401, 95% CI: 0.047 to 0.665, *p* = 0.028, q = 0.176), peak hip power (*r* = 0.389, 95% CI: 0.033 to 0.657, *p* = 0.034, q = 0.176), and ankle joint work (*r* = 0.457, 95% CI: 0.115 to 0.702, *p* = 0.011, q = 0.118). These findings should be interpreted as supportive exploratory evidence because they did not survive FDR correction. Eccentric-phase joint kinetic variables, concentric-phase knee kinetic variables, concentric-phase peak ankle moment, concentric-phase peak ankle power, and joint stiffness variables were not significantly associated with jump height in this sample.

**Table 3:** Correlations between hip, knee, and ankle joint kinetics and jump height

Variable	*r* (95% CI)	*p*	q
Eccentric phase			
Peak hip moment	0.336 (-0.027, 0.621)	0.069	0.291
Peak hip power	-0.081 (-0.429, 0.287)	0.670	0.946
Hip joint work	-0.017 (-0.375, 0.345)	0.927	0.967
Peak knee moment	0.235 (-0.137, 0.549)	0.211	0.443
Peak knee power	-0.008 (-0.367, 0.353)	0.967	0.967
Knee joint work	0.051 (-0.315, 0.404)	0.790	0.967
Peak ankle moment	0.040 (-0.325, 0.395)	0.833	0.967
Peak ankle power	-0.076 (-0.425, 0.292)	0.690	0.946
Ankle joint work	0.018 (-0.345, 0.376)	0.926	0.967
Concentric phase			
Peak hip moment	0.401 (0.047, 0.665)	0.028	0.176
Peak hip power	0.389 (0.033, 0.657)	0.034	0.176
Hip joint work	0.610 (0.320, 0.795)	< 0.001	0.007
Peak knee moment	0.303 (-0.064, 0.598)	0.104	0.311
Peak knee power	0.216 (-0.157, 0.535)	0.252	0.481
Knee joint work	0.262 (-0.108, 0.569)	0.162	0.377
Peak ankle moment	0.068 (-0.300, 0.418)	0.721	0.946
Peak ankle power	0.279 (-0.090, 0.581)	0.135	0.355
Ankle joint work	0.457 (0.115, 0.702)	0.011	0.118
Joint stiffness			
Hip stiffness	0.304 (-0.063, 0.599)	0.102	0.311
Knee stiffness	0.202 (-0.171, 0.524)	0.285	0.499
Ankle stiffness	0.161 (-0.211, 0.493)	0.394	0.637

Confidence intervals for r were calculated using Fisher’s z transformation. Box-Cox transformed variables are reported in Additional file 1

p values are uncorrected; q values are Benjamini-Hochberg FDR-adjusted values across the 21 correlation analyses

## Discussion

The main finding of this study was that the strongest and most statistically robust association was concentrated in concentric-phase hip joint work. Before FDR correction, greater depth-jump height was accompanied by a broader pattern involving concentric-phase hip kinetic variables and ankle joint work. After correction for multiple comparisons, however, only hip joint work during the concentric phase remained significant in both the descriptive median-split comparison and the continuous correlation analysis. The continuous analysis should be regarded as the primary source of evidence because it preserves the full range of jump-height values and does not create outcome-derived categories. The median-split results are presented only to illustrate the magnitude and distribution of differences between relatively higher and lower performers and should not be interpreted as independent confirmation of the correlation. Because the design was cross-sectional, all findings represent biomechanical associations rather than causal training effects.

The absence of significant eccentric-phase associations suggests that the kinetic variables measured during landing and braking were less sensitive to differences in jump height than the variables measured during propulsion. Hunter and Marshall [33] showed that vertical jump technique can be modified by training-related changes in power and flexibility, which supports the view that jump performance is shaped by the coordination of multiple mechanical factors. Tendon and muscle behavior during stretch loading may influence how energy is stored and transmitted before take-off [34]. The stretch-shortening cycle also depends on how effectively the neuromuscular system couples eccentric loading with concentric force production [35]. In the present study, however, the decisive between-group differences appeared after the transition into the concentric phase rather than during the eccentric phase itself, suggesting that the ability to convert braking into upward propulsion may be more informative than isolated eccentric-phase peak values for explaining depth-jump height.

The uncorrected differences in concentric-phase peak hip moment and peak hip power provide supportive context for the hip-work finding. Bobbert et al. [17] argued that countermovement-jump performance benefits from effective joint-moment generation during propulsion. Cleather et al. [36] also emphasized that intersegmental moment patterns are closely related to explosive jumping tasks, and related studies have shown that task goals and lower-limb intersegmental dynamics can alter jump biomechanics [37, 38]. In the present study, peak hip moment and peak hip power were higher in HJG and positively associated with jump height before FDR correction, suggesting that larger net hip moments and power may contribute to the observed hip-work association. However, because these peak variables did not survive FDR correction, they should be interpreted as supportive exploratory evidence rather than statistically robust independent findings.

This distinction between peak variables and joint work is important. Peak moment and peak power are instantaneous values and may be sensitive to movement timing, filtering, and trial-to-trial variation. Joint work is the time integral of net joint power, with joint power determined by the product of net joint moment and joint angular velocity. Consequently, greater concentric hip work may arise from a larger net hip moment, greater angular velocity, a larger hip-extension excursion, a longer propulsive duration, or a combination of these factors. Because countermovement depth, joint range of motion, and ground contact time were not standardized, the greater hip work observed in higher jumpers cannot be attributed solely to greater hip-extensor force-production capacity or interpreted simply as “better hip output.”

Among the variables examined, joint work therefore provided the most robust integrated mechanical association with depth-jump height. Concentric-phase hip joint work was greater in HJG than in LJG and showed the strongest continuous correlation with jump height. Marshall and Moran [18] reported that biomechanical variables associated with jump height can differ between cross-sectional and training contexts, and Vanezis and Lees [19] showed that good and poor vertical-jump performers differ in lower-limb mechanical characteristics. The present results extend this work to male collegiate volleyball players by identifying concentric-phase hip joint work as the most robust lower-limb kinetic correlate among the variables examined. This finding indicates that cumulative net mechanical energy at the hip was more consistently associated with performance than isolated peak values, but it does not identify the specific muscular, kinematic, or temporal mechanism responsible for the association.

Ankle joint work should be interpreted more cautiously. Although peak ankle moment and peak ankle power were not significantly related to jump height, ankle joint work during the concentric phase was higher in HJG and positively correlated with jump height before FDR correction. This pattern suggests that the ankle may provide supportive positive work during take-off, but the evidence was not as robust as that for hip joint work. Augustsson et al. [39] showed that landing depth can influence knee kinematics during drop-jump landings, and Moran and Wallace [21] reported that the relative contribution of the hip, knee, and ankle can vary with knee-joint range of motion. The hip-dominant association observed here may therefore partly reflect the self-selected countermovement and joint-range-of-motion strategies used by individual athletes. The non-significant knee findings should not be interpreted as evidence that the knee is unimportant; knee contribution may have been less variable in this relatively homogeneous sample or may depend more on coordination timing and interaction with the hip and ankle than on isolated peak kinetic variables.

The lack of significant findings for joint stiffness is also meaningful. Stiffness is commonly used to evaluate reactive strength and stretch-shortening-cycle function. Kipp et al. [25] identified mechanical determinants of the reactive strength index during drop jumps, highlighting the value of stiffness-related variables in fast rebound tasks. However, Kalkhoven and Watsford [40] reported that the relationship between mechanical stiffness and performance markers is not always consistent across athletic tasks. Laffaye et al. [41] found that greater jumping expertise does not necessarily coincide with higher lower-limb stiffness. Jidovtseff et al. [22] also suggested that jumping strategy can alter kinetic and kinematic variables in ways that complicate the interpretation of stiffness. The present results are consistent with the view that joint stiffness may be less informative when athletes are allowed to use a larger countermovement and focus primarily on maximizing jump height.

From a practical standpoint, the findings are most applicable to male collegiate volleyball players with training and competitive backgrounds similar to those studied. In laboratories or performance programs with three-dimensional motion analysis, practitioners may monitor concentric hip work alongside jump height, ground contact time, and joint range of motion during repeated assessments. For example, a within-athlete reduction in jump height accompanied by lower hip work may prompt further evaluation of fatigue, technique, or strength status, whereas a similar jump height produced with a different distribution of hip, knee, and ankle work may indicate a change in movement strategy. Concentric hip work may also be used as a hypothesis-generating outcome when evaluating hip-extension strength or plyometric interventions. However, no diagnostic threshold or causal training prescription can be derived from the present data, and pre-post longitudinal testing is required before changes in hip work can be interpreted as a mechanism of performance improvement.

### Limitations

This study has several limitations. First, the sample consisted of Chinese male collegiate volleyball players who held at least National Second-Class Athlete certification and had provincial-level competition experience. The findings should therefore not be directly generalized to female athletes, youth athletes, athletes at substantially different competitive levels, or athletes from other sports. Although a minimum competitive standard was specified, participants were not stratified by certification tier or by the type and extent of their competition experience; residual heterogeneity in competitive background may have influenced the observed biomechanical patterns. Second, the median split provided a descriptive comparison between athletes with relatively higher and lower jump height but reduced the information contained in the continuous outcome. The continuous correlation analysis across the full sample is therefore the primary basis for interpretation. Third, the sample size was modest. A sensitivity power analysis indicated that the available sample could detect a large between-group effect of approximately Cohen’s d = 1.06 and a Pearson correlation of approximately |r| = 0.492 with 80% power at alpha = 0.05; smaller but potentially meaningful effects may have been missed. Fourth, the fixed 30-cm drop height and hands-on-hips condition improved standardization but reduced ecological similarity to volleyball actions involving arm swing, approach steps, repeated contacts, or fatigue. Fifth, ground contact time, countermovement depth, phase duration, and hip, knee, and ankle ranges of motion were not standardized. Because joint work depends on net joint moment, angular velocity, angular excursion, and integration time, individual movement strategies may have influenced the calculated joint-work values. Finally, the study did not include electromyographic activity, tendon behavior, trunk mechanics, or detailed inter-joint coordination timing. Future studies should combine synchronized kinetic and kinematic analyses with electromyography and other neuromuscular measurements to determine whether the observed work differences reflect muscle-activation patterns, net moment production, range of motion, phase duration, or their interaction. Longitudinal intervention studies are also required to test whether modifying these characteristics changes depth-jump performance.

## Conclusion

The most robust finding was that, in this sample of male collegiate volleyball players, greater depth-jump height was associated with greater hip joint work during the concentric propulsive phase, and this relationship remained significant after correction for multiple comparisons. Concentric-phase peak hip moment, peak hip power, and ankle joint work showed supportive uncorrected differences or associations, but these results did not survive FDR correction and should therefore be interpreted as exploratory. Eccentric-phase joint kinetic variables, concentric-phase knee kinetic variables, peak ankle moment, peak ankle power, and hip, knee, and ankle joint stiffness were not significantly associated with jump height in this sample. Concentric hip work may therefore serve as an associative biomechanical marker for monitoring and as a candidate variable for longitudinal study. The present cross-sectional results do not establish whether increasing hip joint work would improve depth-jump height or whether athletes who already jump higher simply use a movement strategy that produces greater hip work.

## Supplementary Information

Additional file 1. Supplementary statistical workbook (.xlsx). Participant-level analysis dataset and supplementary statistical results, including normality tests, Box-Cox transformation records, group-comparison results with FDR-adjusted q values, correlation results with 95% confidence intervals and FDR-adjusted q values, sensitivity power analysis, and jump-height group summary.

Additional file 2.

## Data Availability

The datasets generated and/or analyzed during the current study are included in this published article and its supplementary information files.

## Abbreviations

CI: Confidence interval
CWD: Concentric work done
EWD: Eccentric work done
FDR: False discovery rate
HJG: Figh-jump group
LJG: Low-jump group
